# Physician-patient boundaries in palliative care

**DOI:** 10.1186/s12904-023-01161-0

**Published:** 2023-04-13

**Authors:** Chong Yao Ho, Nicole-Ann Lim, Nur Diana Abdul Rahman, Min Chiam, Jamie Xuelian Zhou, Gillian Li Gek Phua, Eng Koon Ong, Crystal Lim, Anupama Roy Chowdhury, Lalit Kumar Radha Krishna

**Affiliations:** 1 Yong Loo Lin School of Medicine, National University of Singapore, 1E Kent Ridge Road, NUHS Tower Block, Level 11, Singapore, 119228 Singapore; 2 Division of Supportive and Palliative Care, National Cancer Centre Singapore, Level 4, 11 Hospital Crescent, Singapore, 169610 Singapore; 3 Division of Cancer Education, National Cancer Centre Singapore, Level 4, 11 Hospital Crescent, Singapore, 169610 Singapore; 4 Medical Social Services, Singapore General Hospital, 16 College Road, Block 3 Level 1, Singapore, 169854 Singapore; 5 Palliative Care Institute Liverpool, Cancer Research Centre, University of Liverpool, 200 London Road, Liverpool, L3 9TA UK; 6 Duke-NUS Medical School, National University of Singapore, 8 College Rd, Singapore, 169857 Singapore; 7 Lien Centre for Palliative Care, Duke-NUS Medical School, National University of Singapore, 8 College Rd, Singapore, 169857 Singapore; 8 The Palliative Care Centre for Excellence in Research and Education, PalC, PalC c/o Dover Park Hospice, 10 Jalan Tan Tock Seng, 308436 Singapore, Singapore; 9 Department of Geriatric Medicine, Singapore General Hospital, 16 College Road, Block 3 Level 1, 169854 Singapore, Singapore

**Keywords:** Boundary-crossings, Palliative care, Physician-patient relationship, Doctor-patient relationship, Boundaries, Professional identity formation, Personhood, Professionalism

## Abstract

**Background:**

Nurturing effective physician-patient relationships is essential to the provision of patient-centred care. Palliative care physicians may apply boundary-crossings or breaches in professional standards to nurture effective physician-patient relationships. Being highly individualized and shaped by the physician’s narratives, clinical experience, and contextual considerations, boundary-crossings are susceptible to ethical and professional violations. To better appreciate this concept, we employ the Ring Theory of Personhood (RToP) to map the effects of boundary-crossings on the physician’s belief systems.

**Methods:**

As part of the Tool Design SEBA methodology, a Systematic Evidence-Based Approach (SEBA) guided systematic scoping review was employed to guide the design of a semi-structured interview questionnaire with palliative care physicians. The transcripts were simultaneously content and thematically analysed. The themes and categories identified were combined using the Jigsaw Perspective and the resulting domains formed the basis for the discussion.

**Results:**

The domains identified from the 12 semi-structured interviews were catalysts and boundary-crossings. Boundary-crossings attempt to address threats to a physician’s belief systems (catalysts) and are highly individualized. Employ of boundary-crossings depend on the physician’s sensitivity to these ‘catalysts’, their judgement and willingness to act, and their ability to balance various considerations and reflect on their actions and their ramifications. These experiences reshape belief systems, understandings of boundary-crossings and may influence decision-making and practice, underscoring the potential for greater professional breaches when unchecked.

**Conclusion:**

Underlining its longitudinal effects, the Krishna Model underscores the importance of longitudinal support, assessment and oversight of palliative care physicians, and lays the foundation for a RToP-based tool to be employed within portfolios.

**Supplementary Information:**

The online version contains supplementary material available at 10.1186/s12904-023-01161-0.

## Background

Caring for patients with terminal illnesses relies on the nurturing of respectful, open, trusting, and enduring connections between physician and patient [1]. In such complex and often difficult circumstances, maintaining the physician-patient relationship places unique demands upon the physician. Amidst evolving conditions, changing care goals and demands and difficult discussions about prognosis, care, and treatment plans, some physicians opt for “deliberate departures from customary practice to benefit their patients” [2].

These *boundary-crossings* often breach parameters that describe the mutually understood, unspoken, physical, emotional, role-related, time-related and physical limits of a fiduciary relationship between the trusting patient and the caring physician or provider [3–5]. The complex and personalized nature of physician, patient, sociocultural, clinical, contextual, psycho-emotional, existential and relational considerations see attempts at boundary-crossings occupy a range of practices including the sharing of “personal disclosure, explore common ground and shared interests, and share humorous exchanges” [6] to infringements on personal, moral, ethical and practice standards [1]. With unique and diverse considerations associated with each boundary-crossing, few physicians are adequately equipped to draw effective physician-patient boundaries. This may impact clinical objectivity, and result in moral distress, burnout and greater ethical and professional violations [2–5, 7].

By acknowledging that palliative care offers an ideal setting to examine boundary-crossings, a study to examine the lived experiences of these physicians and determine the nature, features and impact of boundary-crossings is proposed [8–11].

### Examining the concept of boundary-crossings

Informed by Gutheil and Gabbard [12], Vig and Foglia [2] differentiate boundary-crossings from egregious boundary-violations such as sexual misconduct and financial exploitation by characterising boundary-crossing as “harmless or benign, sometimes beneficial departure from customary or traditional clinical practice”. Whilst this characterization is increasingly contentious, this definition is selected given its wide use in current practice.

To study boundary-crossings as a sociocultural construct, a holistic perspective is adopted. This includes consideration of the physician’s regnant practice, social, cultural, familial, relational, existential, and clinical considerations (henceforth narratives); personal values, beliefs, and principles (henceforth belief system); clinical experiences, knowledge, skills and competencies (henceforth clinical insights), regnant practice, social, cultural, familial, relational, existential, and clinical considerations (henceforth contextual considerations); and moral, ethical compass, judgement and attitudes towards the employ of boundary-crossings [8, 13, 14].

Interactions between the belief system and the physician’s narratives, clinical insights and contextual considerations are also scrutinized on the premise that the physician’s practice, conduct, thinking, and patient care are informed by their belief system. Therefore, appreciating how these various experiences influence the physician’s belief system would improve the assessment, guidance of practice and direct timely, appropriate, personalized and longitudinal support to physicians. With recent reviews suggesting that interactions between the belief system and physician’s narratives, clinical insights and contextual considerations may be mapped using the Radha Krishna and Alsuwaigh [15]’s Ring Theory of Personhood (RToP) [13, 16–21], we adopt the RToP to study current data on boundary-crossings.

### The ring theory of personhood

Use of the RToP premises that a physician’s belief system is informed by their concepts of identity and personhood. Thus, mapping changes in self-concepts of personhood would highlight shifts in the belief system. The RToP suggests that personhood is comprised of the Innate, Individual, Relational, and Societal domains [22]. The Innate Ring encompasses the physician’s Divine connections and/or their genetic propensity of being human [15]. The Individual Ring encompasses their conscious function which are manifested in the physician’s thinking, decision making, actions and conduct. The Relational Ring comprises important personal relationships while the Societal Ring consists of the physician’s societal and professional obligations.

When new experiences, beliefs, values, principles, insights, and reflections are consistent with regnant belief system within the rings, there is ‘resonance’ [17, 19, 21, 23–25]. When the contents of the belief system are reprioritized to better fit with practical considerations - ‘synchrony’ is created. Conflict between current values, beliefs, and principles and those being introduced within one of the rings, creates ‘disharmony’ whilst ‘dyssynchrony’ occurs when conflicts occur between rings [19, 23]. Adaptations to resonance, synchrony, disharmony and dyssynchrony shape self-concepts of identity and personhood.

## Methods

To determine, “how boundary-crossings impact palliative care physicians’ practice and identities?”, semi-structured interviews were proposed to capture the often complex ethical and moral deliberations that shape physician-patient interactions, their practice, and decision-making processes [26, 27].

### The tool design SEBA methodology

**Fig. 1:**
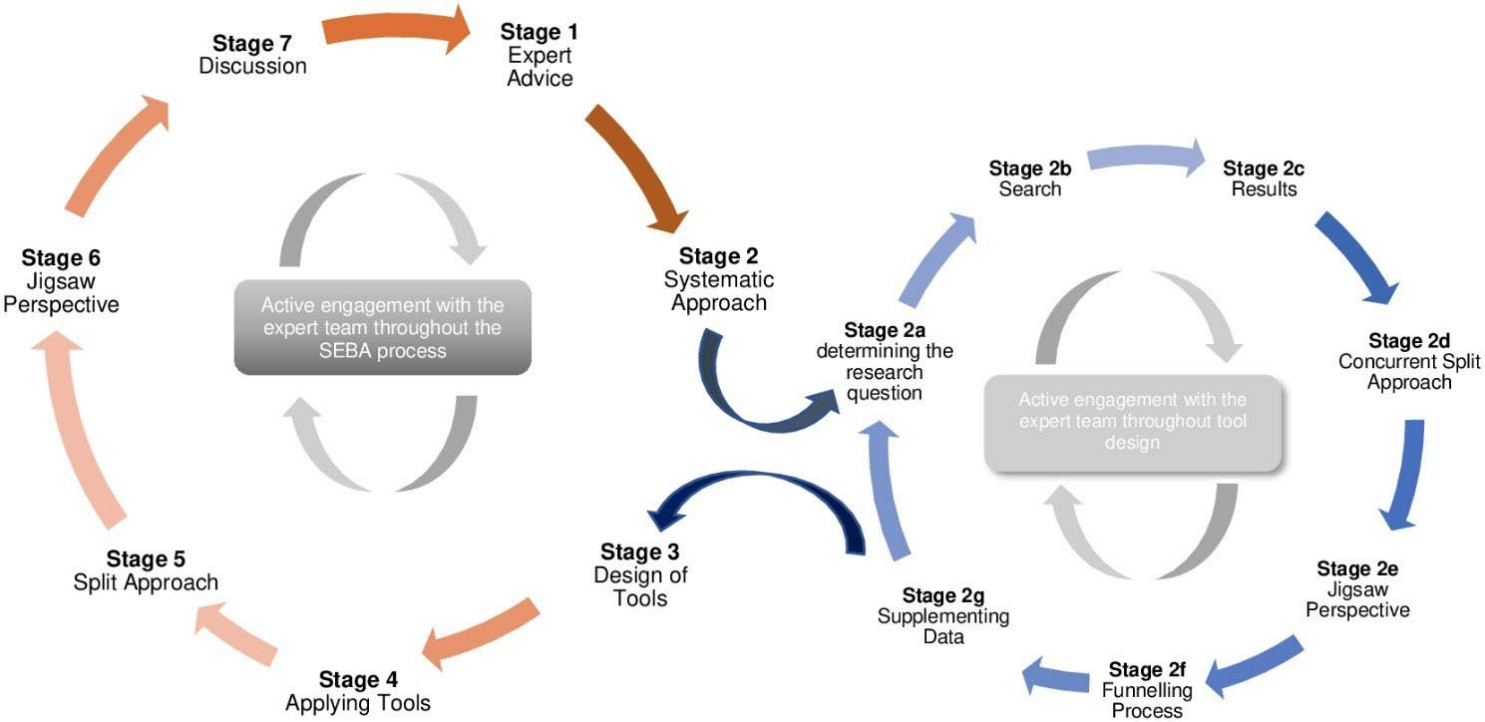
The Tool Design SEBA Process

Adapting Krishna’s Systematic Evidence Based Approach (SEBA), the two-staged Tool Design SEBA methodology (Fig. 1) was used [28–32]. In the first stage, a systematic scoping review was employed to guide the design of the tool. In the second stage,the data accrued from its application was analysed.

#### Stage 1. expert advice

An expert team consisting of a medical librarian from the Yong Loo Lin School of Medicine (YLLSoM) and local educational experts and clinicians at the National Cancer Centre Singapore (NCCS), the Palliative Care Institute Liverpool, YLLSoM and Duke-NUS Medical School ensured a balanced, accountable and trustworthy evaluation of each stage.

#### Stage 2: systematic approach

Having posited that boundary-crossings are evolving and context-specific, the research and expert teams focused on the need to create, apply, and analyse the data accrued from evidence-based semi-structured interview questionnaires. A scoping review was thus conducted to better understand existing literature on boundary crossings in palliative care (Appendix A).

#### Stage 3. design and ethics approval of semi-structured interview

Data from the scoping review was then used to design the semi-structured interview questionnaire (Appendix B).

Ethics approval (2021/2176) was obtained from the SingHealth Centralised Institutional Review Board.

#### Stage 4. application semi-structured interviews

Eligible participants were palliative care physicians at the Division of Supportive and Palliative Care (DSPC) at the National Cancer Centre Singapore (NCCS).

The semi-structured interviews were conducted via video conferencing, with resident physicians, associate consultants, consultants and senior consultants at the Division of Supportive and Palliative Care (DSPC) at the National Cancer Centre Singapore (NCCS), between July and August 2021. Purposive sampling was conducted and email invitations containing participant information sheets, study information, and details on the nature, duration and aims of the audio-recorded interviews. The invitations stressed participants’ anonymity in the audio-recorded semi-structured interviews and highlighted their right to withdraw from the study at any point and without prejudice. Two trained members of the research team (ASIL and MC) sought verbal and written consent before conducting the interviews which lasted about 45 minutes. These interviews took place in quiet offices to ensure privacy and facilitate an in-depth exploration of personal beliefs, experiences, and practices. Audio recordings were transcribed verbatim using the NVivo 12 Software, anonymised, and ‘member checked’.

#### Stage 5: split approach

##### Thematic analysis

Braun and Clarke [33]’s approach to thematic analysis was employed to independently construct ‘codes’ from the ‘surface’ meaning of the transcripts. Overseen by the expert team, the ‘codes’ were discussed online and categorized into groups. A coding framework and code book was agreed upon using ‘negotiated consensual validation’. The remaining transcripts were independently coded and categorized into sub-themes and themes.

##### Directed content analysis

Hsieh and Shannon’s approach to directed content analysis was also employed. Chan et al. [23] and Kuek et al. [19]’s studies, “*Extending the Ring Theory of Personhood to the Care of Dying Patients in Intensive Care Units*” and “*The Impact of Caring for Dying Patients in Intensive Care Units on a Physician’s Personhood: A Systematic Scoping Review*”, provided the categories.

#### Stage 6 of SEBA: jigsaw perspective

The Jigsaw Perspective employed Phases 4 to 6 of France et al. [34]’s adaptation of Noblit and Hare [35]’s seven phases of meta-ethnography [36] to combine overlapping areas within the themes and categories.

## Results

A total of 13 palliative physicians, labelled p1 to p13, comprising five senior consultants, three consultants and two associate consultants and three resident physicians, were interviewed.

Thematic analysis of the data revealed four themes: motivations, boundary-crossings, reflections and adaptations. Directed content analysis revealed three categories: belief system, catalysts for boundary-crossings and the impact of boundary-crossings along the RToP.

The Jigsaw Perspective gave rise to two final domains: (1) catalysts and (2) boundary-crossings.

### Domain 1. catalysts

Beyond traditional notions of boundary-crossings being confined to specific physician-patient relationships, catalysts can present as general threats to overall care provisions. Physicians p1, p3, p5, p7 and p9 breached personal and relational beliefs and responsibilities to sustain their palliative care involvement and their contribution to a field struggling with shortages (Table 1).

**Table 1:** Regnant Belief System and Catalysts through the Lens of the RToP

Rings of the RToP	Regnant Beliefs	Catalysts
Innate	*“[caring for a dying patient] I was playing some hymns, and then suddenly my change of heart was just like that (to be a palliative care physician). It’s very strong… very certain to me.”* (p3)	*“There’s a phase that you go through where there’s hopelessness and there’s suffering because patients don’t see a way out, and we can’t seem to help them with their helplessness. So, that leads some to some spiritual questioning.”* (p7)
Individual	*“You really can treat the patient holistically and really get to know their whole family. You really journey with them as a whole right till the end and I think it’s really a privilege… so, that’s really what put me in palliative.”* (p1)	*“Sometimes, when you are so caught up with caring for a patient, sometimes it’s hard to take leave. Because you are like, oh no, I’m leaving my patient (in the) ward. Can the other team cope?”* (p1)
Relational		*“Staying very, very late and going in unnecessarily on weekends and things like that and just scrutinising everything that you do…so . you’re thinking about your patients when you’re at home and you’re thinking about your child when you’re at work.”* (p11)
Societal		*“So a lot of times, especially now with COVID, and you don’t get to travel, and even when you take leave, and you’re at home, people (from work) know that you’re actually physically around and you can always constantly be called.”* (p4)

### Domain 2. boundary-crossings

Recognizing a catalyst, determining its significance, balancing its potential risks and benefits of attempts to ameliorate this threat gives way to a decision to apply boundary-crossings. Boundary-crossings maybe adaptive or proactive (Table 2).

**Table 2:** Adaptive and Proactive Boundary-Crossings

Rings of the RToP	Adaptive	Proactive
Innate	Boundary crossed - evaluating prevailing beliefs *“There’s a phase that you go through where there’s hopelessness and there’s suffering because patients don’t see a way out, and we can’t seem to help them with their helplessness. So, that leads some to some spiritual questioning.”* (p7)	Being asked to pray for cure/healing by a terminally ill patient, p7 felt like a boundary was crossed - providing ‘false’ hope to the patient though ‘cure/healing’ was still possible *“I do have to think through it… what are the landmines and all that? What do I pray for and what do I not pray for? Because for us, praying for healing, you are in the middle of difficult grounds. So, I have to set up things that generally I feel I can pray for.”* (p7)
Individual	Boundary crossed - compromising personal interest *“Over time, you learn to set your own boundaries so you can continue working happily.”* (p1)	Boundary crossed - Being able to relate to a patient on a personal level can compromise professional detachment and affect judgement *“When you see a young patient that is your age… you’re definitely going to try and help the patient even harder, do even more things for the patient.”* (p10)
Relational	Boundary crossed – causing distress *“Initially, I brought work home. I realised over the years that my family cannot take all these sad stories… it distresses them.”* (p3)	Boundary crossed - Allowing professional duties to trump personal relational responsibilities *“Staying very, very late and going in unnecessarily on weekends and things like that and just scrutinising everything that you do… It’s also the mom guilt, that you’re balancing work and child and you’re thinking about your patients when you’re at home and you’re thinking about your child when you’re at work and things like that.”* (p11)
Societal	Boundary crossed - Creating distance *“I don’t think you need to give 100% all the time. You can just give enough… because I think if you give your all, all the time then there’s nothing left to give.”* (p8)	Boundary crossed- losing independence Seeking multi-professional perspectives can constrain professional development and practice *“We encourage people to voice their opinions and thoughts for the patient’s management. So, even in my own team, I can sometimes have three different opinions from people of different seniority.”* (p5)
Multiple Rings	Reflecting on current practice Individual and Societal Rings *“I start taking measures or strategies to relax and take time off.”* (p1)	Reflecting on current practice Individual and Societal Rings *I didn’t know whether I was deciding as a doctor or as a friend. And so, it became very tricky… And I didn’t know whether that (boundary-crossing) would be good for the patient and their family as well.” (p5)* Individual, Relational and Societal Rings *“I continued contacting her. We continued messaging each other and we’d almost chat like friends. I know it was very blurred and sometimes I was a bit confused.”* (p3)

Adaptive boundary-crossings or adaptations are changes to the belief system in response to the effects of catalyst. Proactive boundary-crossings occur when the physician acts pre-emptively to negate the effects of a catalyst.

The decision on whether adaptive or proactive boundary-crossings are to be employed is determined by the physician’s self-awareness, clinical insights and reflections. The impact of boundary-crossings includes ‘ring-fencing’ an experience as a guide or warning against future boundary-crossings or similar situations. This is exemplified by Physician p5’s ‘ring-fencing’ the loss of a patient with whom they shared close personal ties to as a guide for future conduct and boundaries for relationships with patients. Physicians p1, p6, p9 and p11, on the other hand, ‘compartmentalized’ their ‘difficult’ experience in order to continue their ‘usual’ practices, unencumbered by the need for changes to their practice.

#### Stage 7 of SEBA: discussion

Our research addresses several misconceptions. To begin, boundary-crossing are not limited to preserving physician-patient relationships but may be focused on the desire to sustain a career in palliative care. In the latter, boundary-crossings are deliberate departures from understood, unspoken, physical, emotional, role-related, time-related, and physical limits of a role as a palliative care physician. Familial, relational and personal roles, physical, emotional, existential considerations and ‘working time’ directives are compromised as a result. Unsurprisingly boundary-crossings can undermine clinical objectivity and result in moral distress, burnout and greater ethical and professional violations [2–5, 7].

Two, it is also unsurprising that data suggests that boundary-crossings are more common than previously believed and often unrecognized in its various forms even amongst experienced palliative care physicians.

Three, the desire to provide holistic care and nurture longitudinal relationships with patients, their families, and caregivers may propagate the perception of boundary-crossing as a ‘benign’ occurrence [4, 37, 38].

Four, the notion that belief systems evolve with increasing ‘boundary-crossing’, precipitating a relaxing of guiding principles that confine practices and creating ‘slippery slopes’ to greater breaches in professional, ethical, personal, moral and legal standards is a concerning finding. Unobserved and unchecked, the possibility of these breaches increases sharply.

Five, the physician’s individualized decision to employ boundary-crossings reflects their sense of identity and its influence on their practice. It is preservation of this identity that guides their ‘sensitivity’ to the presence of catalysts, informs their ‘judgement’ to determine the need to respond to a catalyst, informs their ‘willingness’ and ability to employ these boundary-crossings effectively to advance their objectives and their knowledge on how to ‘balance’ practical and personal considerations with their skills, abilities and experience to address the catalyst and sustain their existing belief systems [28].

#### The Krishna model for boundary crossings

**Fig. 2:**
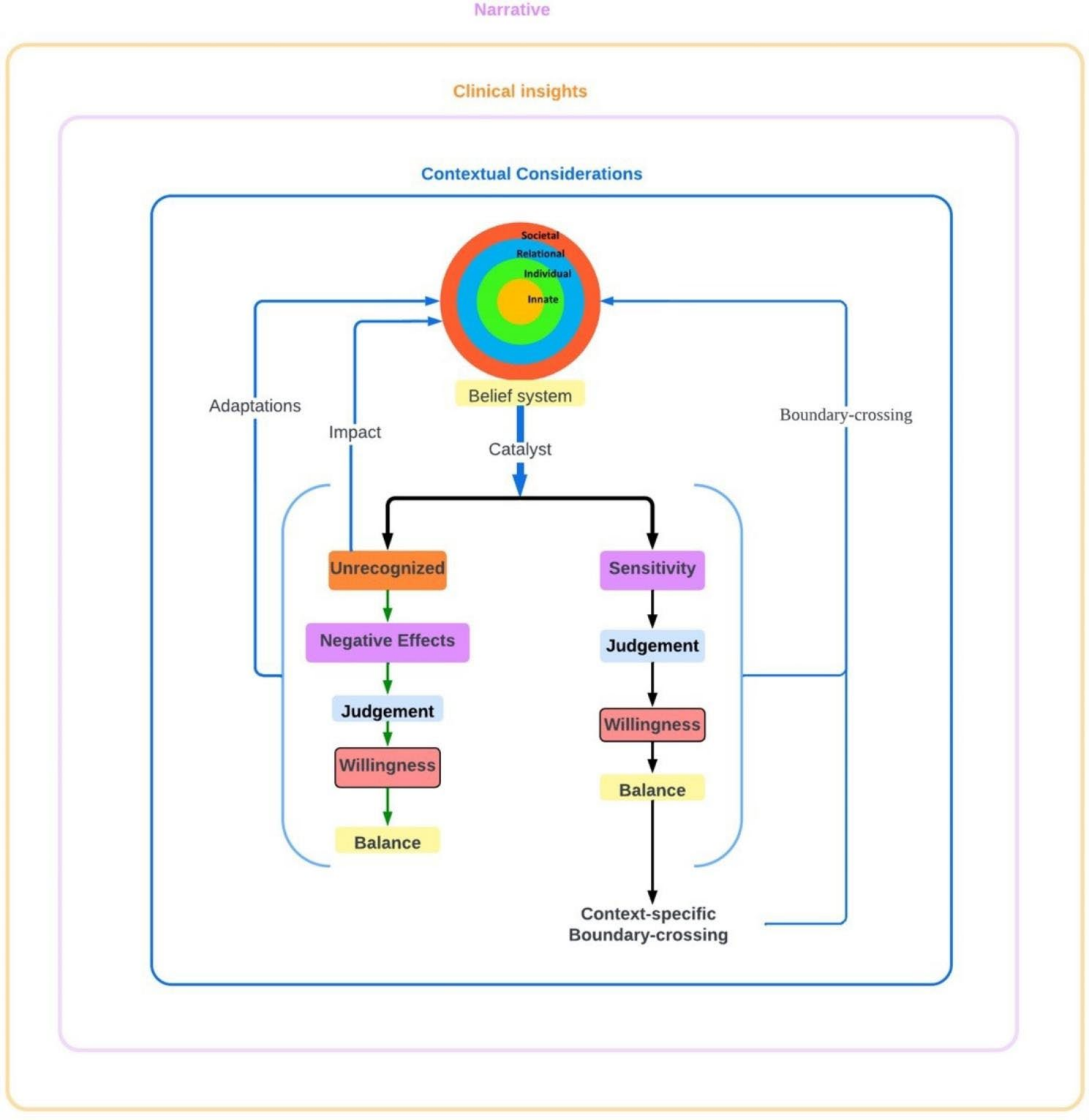
The Krishna Model for Boundary Crossings

These findings allow the proffering of the Krishna Model for boundary-crossings (Fig. 2). This model recognizes the physician’s narrative, contextual considerations and clinical insights in shaping the belief system and the processes behind boundary-crossings. As a result, the belief system depicted by the RToP is encircled by three rings.

**Fig. 3:**
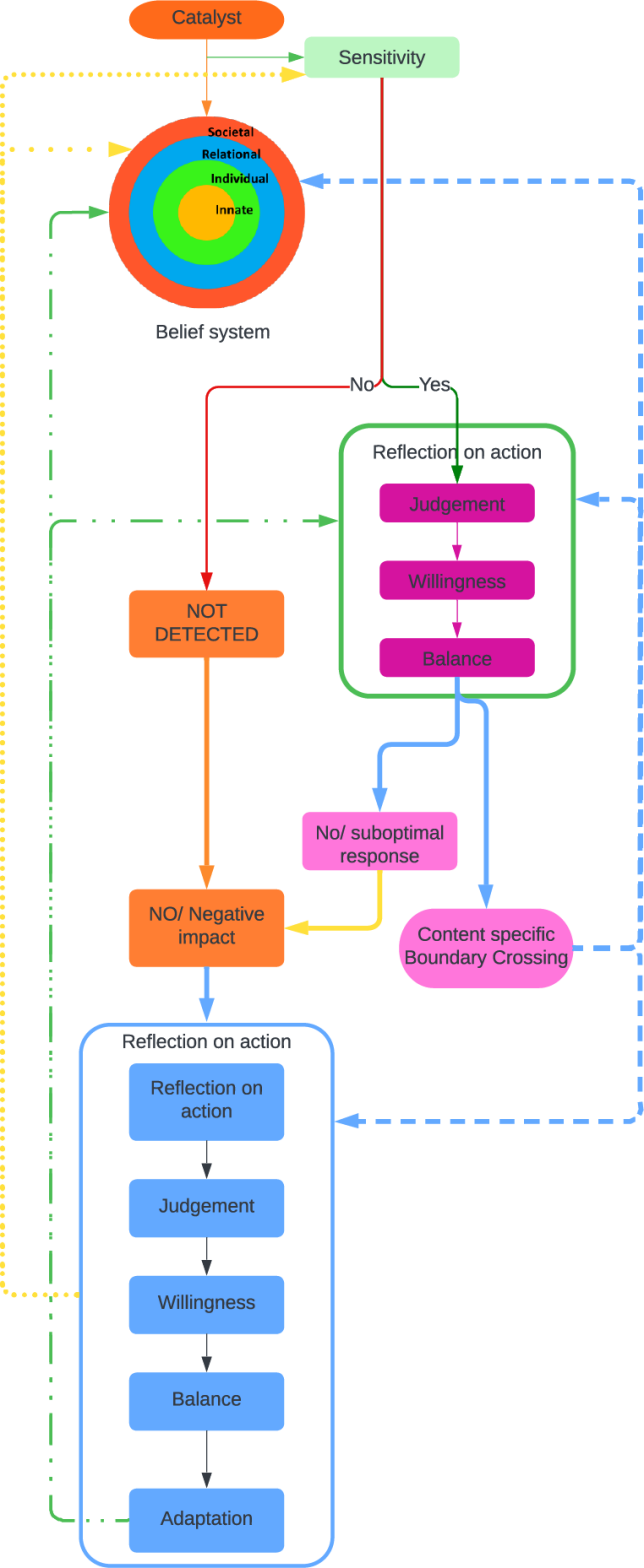
Potential Effects of Boundary Crossings

In this model, boundary-crossings are cast as a means to attenuate the effects of a catalyst. When a catalyst is detected, an internal system of decision-making involving the physician’s ‘sensitivity’, ‘willingness’, ‘judgement’ and ‘balance’ is set in motion and informed by a continuous process of review or ‘reflections-in-action’. This results in either a boundary-crossing or no response. If a catalyst is not detected until after its effects are felt or if the response is suboptimal, reflection-on-action ensues. Similarly, adoption of a boundary-crossings sees reflections-in-action initiated. The insights gained from reflections in both circumstances reshape the belief system (Fig. 3).

It is here that its relevance to clinical practice becomes clear. The frequent presence of boundary-crossings and their impact upon a physician’s belief system, professional identity and patient care underlines the need for access to longitudinal and accessible support. This will help them as they confront boundary-crossings and the closely related issues of moral distress and emotional, ethical and practical issues surrounding care of dying patients [17, 28, 30–32, 39]. This model also echoes concepts behind reflective practice and the teaching of empathy.

These insights underscore the need for longitudinal assessment-driven support of physicians. Consequently, it also underlines the potential for a RToP-based tool, designed to evaluate the physician’s needs, coping and progress. Both considerations coalesce to highlight the need for a portfolio-based program as an additional source of reflection and to guide support and oversight of physicians [40–46].

#### Recommendations

Drawing upon our findings, we propose four measures to support physicians in navigating professional boundaries.

One, incoming junior physicians should be oriented to the concept, prevalence and ill-effects of boundary-crossings and be equipped to identify the risk factors and early signs of boundary violations.

Two, the Krishna Model may be used to facilitate faculty development programs on identifying ‘at risk’ physicians such as those identifying strongly with a saviour complex and differing goals of care which make them especially prone to boundary-crossings [4, 47]. In each case, faculty members should be able to evaluate the physician’s level of self-awareness, willingness to seek help, ability to reprioritize and balance work-life commitments, delegate work to a wider team, and their ability to self-care. These evaluations must be accompanied by individualized assessments of the work environment, the availability and effectiveness of peer, counselling, spiritual, psychological, and psychiatric support and the potential to take ‘time off’ either for a leave of absence or to focus on non-patient facing work.

Such a comprehensive assessment and support mechanism for physicians may be structured around a portfolio-based program [40, 46] and supported by a RToP-based assessment tool. This portfolio-based program echoes Fronek et al. [48]’s calls for “a model of training that can meet multi-level learning needs of practitioners” that “is delivered along the professional life span[ing] and incorporate[ing] external reinforcement, inclusive of peer support and supervision opportunities”. This portfolio-based approach must be supplemented by safe communication channels and a structured mentorship program within a psychologically ‘safe’ practice environment [49, 50].

Three, faculty should be equipped to support physicians with distress tolerance skills such as compassion-based and mindfulness-centred interventions [13, 14, 51], self-soothing relaxation techniques, guided imagery, biofeedback, exercise, and mindfulness [47, 52, 53]. Faculty, too, should have access to assess, coordinate, and supplement their assessment and support with peer, counselling, spiritual, psychological, and psychiatric support programs.

Four, underpinning the efficacy and sustainability of the three recommendations made must be a host organization capable of designing and supporting effective recruitment and training of physicians and faculty, creating, and overseeing the portfolio-based approach and nurturing a supportive and secure work environment.

### Limitations

This study is not without its limitations.

Perhaps prime amongst these is the attempt at establishing causality or linking both events to adaptations to belief systems and sustained changes in professional identities. Neither can be accurately made even amongst senior palliative care physicians within a single tertiary healthcare cluster. Multisite studies across the Southeast Asian context and beyond could provide greater insights and enhance the applicability of these findings.

Other limitations include the use of purposive sampling which draws attention to physicians who already are aware of boundary-crossings and their effects. In addition, although analysis of the interviews was carried out by two investigators through triangulation of data, the omission of member checking is a concern. Additionally, whilst inspired by studies on dignity, professional identity formation, moral distress, empathy, and caring for the dying, use of the RToP to guide the analysis has not been evidenced and should be evaluated further.

## Conclusion

Reframing boundary-crossings invites scrutiny of the often complex psychosocial, financial, practical, cultural and relational settings that occurs within palliative care. This is especially so in Confucian-inspired societies such as China, Singapore, Hong Kong, Taiwan, Korea, and Japan [8–10, 54–57] where the role of the physician is seen as part of the family unit inviting boundary-crossings and highlighting its sociocultural roots. Here focus for future studies should be upon the design and employ of a RToP-based tool [13, 16, 23, 50, 58, 59] and on the experiences of all members of the palliative care multidisciplinary team and healthcare professionals in fields such as intensive care and specialities involved in the treatment of chronic illnesses.

## Electronic supplementary material

Below is the link to the electronic supplementary material.

**Appendix B. Interview Questions**

**Appendix A. SEBA guided in Scoping Reviews**

## Data Availability

The datasets used and/or analysed during the current study are available from the corresponding author on reasonable request.

## List of abbreviations

DSPC: Division of Supportive and Palliative Care (of NCCS)
NCCS: National Cancer Centre Singapore
RToP: Ring Theory of Personhood
SEBA: Systematic Evidence Based Approach
