# Supplementary material for: Physician-patient boundaries in palliative care
Source: BMC Palliat Care. 2023 Apr 13;22:41. doi: 10.1186/s12904-023-01161-0 (PMC10099695; doi:10.1186/s12904-023-01161-0)
Supplement: Supplementary file 1 — Appendix B. Interview Questions [file 12904_2023_1161_MOESM1_ESM.pdf]

Appendix B. Interview Questions

Topic Areas	Questions
**Demographic Questions**	*To start off this interview, we would to understand a bit more about your background in relation to your current career in palliative medicine / medical oncology / radiation oncology.*<br><br>1. Could you share with us what drew you to medicine?<br>2. Were there particular personal values or beliefs that attracted you to this field?<br>3. What drew you to palliative medicine / medical oncology / radiation oncology?<br>4. How long have you been in palliative medicine / medical oncology / radiation oncology?<br>5. What is your day like in the course of your work?
**Memorable Case**	***Guide for Interviewer***<br>• *Start off with discussing a memorable case with the participant before moving on to prompt about each ring of personhood and dyssynchrony if it has not been covered in the case discussion.*<br>• *Questions from the section **'Conflict & Dyssynchrony'** can be incorporated into the discussion at any stage where relevant.)*<br><br>*Thanks for sharing that, we would now like to understand more about any memorable cases you had in the course of your work.*<br><br>6. **Could you share with us a case which was memorable to you or a case which rocked your confidence, beliefs, or faith?**<br>7. **Why was this case memorable to you?**<br>8. **How has this case impacted you?** OR<br>9. **How have you adapted or changed following this case?**<br><br>*(If not already raised, go to questions under the innate / individual / relational / societal rings)*<br>**Possible prompts:**<br>• If the case is not suitable to discuss a specific ring → has there been other cases where…?<br>• Did it affect your perspective on a good life and death? → go to questions under innate ring<br>• How did this case affect your perspective on palliative care/your day-to-day emotions/the way you see yourself/what you value in life? → go to questions under individual ring<br>• How did this case affect your relationships with the people who matter to you? → go to questions under relational ring<br>• How did this case affect your perspective on being a physician / APN? → go to questions under societal ring<br>• If the concept of **'dyssynchrony'** is brought up in discussion of the other rings → Go to questions 8-15 under 'Conflicts & Dyssynchrony'.
**Conflict & Dyssynchrony**	10. **In the case you have highlighted, was there some conflict with what you believed in versus what was expected of you?**

	- For e.g., some doctors we had interviewed struggled with caring for suffering patients as they were questioning whether they were prolonging the dying processes rather than prolonging a good quality of life for the patient.<br>- For e.g., because of your personal beliefs, do you agree with the use of supportive measures that may potentially prolong the dying process rather than improve the quality of life? Or even may potentially shorten life<br><br>11. (Possible Follow-up) →<br>- **How do you feel about this conflict?**<br><br>12. (Effect) → **Did it affect your decision-making or your actions?**<br>13. (Effect) → **How has this conflict affected you?**<br>- For e.g., has this conflict made you feel burnt out, angry, influenced your thoughts about your career, or has the thought of quitting because the conflict was too great to bear crossed your mind?<br>- For e.g., have you had to give up your religion / something important to you that you held for very long because of the conflict?<br>- **how did you deal with this- what helped you find an answer and balance** (Who or what helped you in achieving resolution?)<br>- **Has it changed along the way in the course of your career?**<br><br>14. **What happened after that?**<br>Possible follow-up questions:<br>- How did you adapt to these difficult situations or people?<br>How have you addressed this conflict?<br><br>15. **Have you experienced instances of moral distress in your line of work?**<br>- I.e. when you encounter situations where your personal moral/ethical values were in conflict with the needs, preferences or decisions of the patient, their family, the medical team, or the larger healthcare system?<br><br>**Possible prompts:**<br><br>- Have you witnessed healthcare providers giving **“false hope”** to a patient or family?<br>- Have you participated in care that caused **unnecessary suffering** or compromised the **dignity** of the patient?<br>- Have you witnessed a **violation of a standard of practice or a code of ethics** and not felt sufficiently supported to report the violation?<br>- Have you witnessed a member of your team being **disrespectful or abusive** to a patient, their family, a colleague or junior member?<br>- Have you witnessed a member of your team making **disparaging or demeaning remarks** about a patient, their family, a colleague or junior member behind their backs?<br>- Have you witnessed **low quality of patient care** due to incompetence, poor team communication or lack of provider continuity?<br>- Have you witnessed compromised patient care due to **lack of resources, equipment, bed capacity or administrative support**?<br>- Have you witnessed **stigmatizing social circumstances** or conditions (e.g. alcoholism, drug abuse, homelessness, or obesity) leading to less than optimal care being provided?

	Go to respective rings that were not covered in the case discussion above
**Innate Ring**	*Thank you, I would now like to move on to exploring how caring for dying patients has influenced innate aspects of your personhood in particular your existential or religious beliefs.*<br><br>16. **How do you view life and death?**<br>• For e.g., do you hold the view that life has inherent meaning?<br>• For e.g., could you share with us what you think makes a "good life" or "good death"?<br>17. **Have you always held this view? / Has this concept of life and death always been there or has it changed as you cared for dying patients?**<br>• For e.g., have you always been (against euthanasia)?<br>18. **Are these concepts still evolving?**<br>19. (Based on their personal conception of life / death)<br>• **What are the principles or values that influence the way you think about life and death?**<br>• **Do you think these beliefs influence your decision-making and the actions that you take and if so how?**
**Relational Ring**	*Thank you, I would now like to go on to explore how this case has influenced your relationships with those close to you.*<br><br>20. **How do you view your relationships with people who matter to you?**<br>• For e.g., your family, close friends, or your significant other?<br>• Some doctors / nurses we interviewed said that caring for dying patients makes them distance themselves for fear of loss<br>21. **Has this always been the case or has this changed in the course of caring for dying patients? (If it has changed, why?)**
**Societal Ring**<br>*Professional Relationships*	*Thank you, I would now like to move on to explore how your experiences have shaped your ties with society.*<br>*Thinking about your job in caring for patients at the end of life, those who receive less than a good death.*<br><br>22. **How have these experiences affected how you view your role as a physician/APN?**<br>23. **How have these experiences changed the way you think about your roles and responsibilities as a physician / APN?**<br>• For e.g., do you think you play God extubating patients, stopping feeding, etc.?<br>24. **How does caring for dying patients affect the way you think about your patients?**<br>25. **How does caring for dying patients affect the way you think about your patient's family?**<br>26. **How does caring for dying patients affect the way you think about your colleagues?**<br>27. **How does caring for dying patients affect the way you think about people in society at large?**

**Individual Ring**	*Thank you, I would now like to explore how this case has influenced you as an individual especially in relation to your conscious functions which is your ability to think, talk and act autonomously.*<br><br>28. **What are the skills you possess that you believe are important to have in caring for dying patients?**<br>29. **Have these been shaped by your experiences?**<br>30. **How do you view patients who are unable to express themselves, or who lack mental capacity or volition or those who are unconscious?**<br>• For e.g., do you think they have rights or value? How do you respect them?<br>31. **Has this always been the case or has this changed?**
