# Supplementary material for: Physician-patient boundaries in palliative care
Source: BMC Palliat Care. 2023 Apr 13;22:41. doi: 10.1186/s12904-023-01161-0 (PMC10099695; doi:10.1186/s12904-023-01161-0)
Supplement: Supplementary file 2 — Appendix A. SEBA guided in Scoping Reviews [file 12904_2023_1161_MOESM2_ESM.docx]

**Appendix A.**

**SEBA guided in Scoping Reviews**

**STAGE 2 of SEBA: The Systematic Approach**

At Stage 2 of the systematic approach, the SEBA process further extends from the main SEBA cycle to ensure active engagement throughout the tool design. A SEBA guided in Scoping Reviews (ScR in SEBA) needs to be carried out to ensure a systematic, reproducible research process to guide the design of the research tools. The ScR in SEBA comprises the following elements: 2a) Determining the research question, 2b) Searching, 2c) Results, 2d) Concurrent split approach, 2e) Jigsaw approach, 2f) Funnelling process and 2g) Supplementing data.

**Stage 2a. Determining the research question**

1. Determining the title and background of the review

Ensuring a systematic approach to the synthesis of SSRs in SEBA, the expert team, stakeholders and the research team agreed upon the overall goals of the SSR and the population, context, and concept to be evaluated. The PICOs is featured in Table 1.

1. Identifying the research question

Members of the research team discussed the research question with medical librarians from the medical library at the Yong Loo Lin School of Medicine at the National University of Singapore (NUS), and local clinicians from NCCS, NUS, and Singapore General Hospital. Guided by the Population Concept, Context (PCC) elements of the inclusion criteria, the research question was “*What is known about boundary-crossings in Palliative Care?*”.

1. Inclusion criteria

All grey literature, peer-reviewed articles, narrative reviews, systematic, scoping, and systematic scoping reviews published between 1^st^ January 2000 to 30^th^ June 2022 were included in the PCC (population, concept, and context) inclusion criteria (1, 2).

Table S1. PICOs inclusion and exclusion criteria

| **Boundary Crossings in Palliative Care** | | |
| --- | --- | --- |
|  | Inclusion criteria | Exclusion criteria |
| Population | - Juniors doctors residents, specialists and/or doctors and/or physicians within the clinical, medical, research and/or academic settings - Healthcare personnel and educators in allied health specialities and medicine - Undergraduate and postgraduate medical students | - Allied health specialties such as Pharmacy, Dietetics, Chiropractic, Midwifery, Podiatry, Speech Therapy, Occupational and Physiotherapy, Physician Assistants - Non-medical specialties such as Clinical and Translational Science, Alternative and Traditional Medicine, Veterinary, Dentistry |
| Intervention | - Papers that addressed boundary-crossings in palliative care among junior doctors, residents, specialists and/or doctors and/or physicians and/or medical students within the clinical, medical, research and/or academic settings | - Papers with little detail of boundary-crossing - Papers that evaluated boundary-crossing for purposes other than on palliative care |
| Comparison Outcome | - Types of boundary-crossings and their effects - Impact of boundary-crossing on junior doctors, residents, specialists and/or doctors and/or physicians and/or medical students within the clinical, medical, research and/or academic settings - Motivations when joining palliative care |  |
| Study design | - All study designs including: mixed methods research, meta-analyses, systematic reviews, randomized controlled trials, cohort studies, case-control studies, cross-sectional studies, descriptive papers, grey literature, opinions, letters, commentaries and editorials - Articles in English or translated to English - Year of Publication: 1^st^ January 2000- 30^th^ June 2022 | - Non-English language articles |

**Stage 2b. Searching**

To study the area of interest, the searches were restricted to articles published between 1^st^ January 2001 and 30^th^ June 2022, after considering the study resources and time constraints (3). This also helped ensure the sustainability of this study. In keeping with the SEBA methodology, quantitative, mixed, and qualitative research methodologies were considered. The PICOs format was employed to guide the searching of articles (Table 1). The ten-membered research team carried out independent searches using variations of the terms “boundaries”, “boundaries in palliative care”, and “boundary crossings”.

**Stage 2c. Results**

A total of 591 titles and abstracts were independently reviewed by the research team to identify relevant articles that met the inclusion criteria set out in Table 1. 73 full-text articles were then filtered by the independent reviewers producing their final list of 18 included articles. These lists were discussed at online reviewer meetings. Sandelowski and Barroso (4)’s approach to ‘negotiated consensual validation’ was used to achieve consensus on the final list of articles to be included (Figure 1.)

**Figure S1. The PRISMA flowchart**

Database search:

1^st^ January 2000 to 30^th^ June 2022

Total: 591 articles

Excluded articles based on exclusion criteria

18 articles included

Abstracts reviewed: 591

Full texts reviewed: 73 articles

Excluded non-relevant articles based on title and abstract

**Stage 2d. Concurrent split approach**

The findings of the ScRs in SEBA were analysed. The ScR in SEBA was then subject to concurrent use of Braun and Clarke (5)’s thematic analysis and Hsieh and Shannon (6)’s directed content analysis (Split Approach). The Split Approach is part of the SEBA methodology and ensures a comprehensive, reproducible, transparent analysis (7-9).

**Stage 2e. Jigsaw approach**

The Jigsaw Perspective (10, 11) sees the findings of both reviews combined. Here, overlaps and similarities between the themes and categories were combined to create themes/categories. The themes and subthemes would be compared with the categories and subcategories identified, and similarities were verified by comparing the codes contained within them. Individual subthemes and subcategories were combined if they were complementary in nature.

**Stage 2f. The Funnelling Process**

The Funnelling Process combines the themes/categories from both ScRs in SEBA using Phases three to five of France, Uny (12)’s approach. The resultant Funnelled themes/categories were then reviewed by the expert team, forming the basis of the tool design.

**Stage 2g. Supplementing data**

To contextualise the Funnelled themes/categories the expert and research teams elected to supplement these findings with results from current studies on the key aspects of boundary-crossings. This saw the inclusion of issues and influences of boundaries in the palliative care (13-16).

The research team also considered Sarraf-Yazdi et al (17)’s recent and comprehensive review of PIF in medical schools entitled “*A Scoping Review of Professional Identity Formation in Undergraduate Medical Education*” to contextualise the findings.

**References**

1. Peters M, Godfrey C, McInerney P, Soares C, Khalil H, Parker D. The Joanna Briggs Institute reviewers' manual 2015: methodology for JBI scoping reviews2015 April 29, 2019. Available from: <http://joannabriggs.org/assets/docs/sumari/Reviewers-Manual_Methodology-for-JBI-Scoping-Reviews_2015_v1.pdf>.

2. Peters MD, Godfrey CM, Khalil H, McInerney P, Parker D, Soares CB. Guidance for conducting systematic scoping reviews. Int J Evid Based Healthc. 2015;13(3):141-6.

3. Pham MT, Rajić A, Greig JD, Sargeant JM, Papadopoulos A, McEwen SA. A scoping review of scoping reviews: advancing the approach and enhancing the consistency. Res Synth Methods. 2014;5(4):371-85.

4. Sandelowski M BJ. Handbook for synthesizing qualitative research. New York: Springer; 2007.

5. Braun V, Clarke V. Using thematic analysis in psychology. . Qualitative Research in Psychology 2006;3(2):77-101.

6. Hsieh H-F, Shannon SE. Three Approaches to Qualitative Content Analysis. Qualitative Health Research. 2005;15(9):1277-88.

7. Kamal NHA, Tan LHE, Wong RSM, Ong RRS, Seow REW, Loh EKY, et al. Enhancing education in Palliative Medicine: the role of Systematic Scoping Reviews. Palliative Medicine & Care: Open Access. 2020;7(1):1-11.

8. Ong RRS, Seow REW, Wong RSM. A Systematic Scoping Review of Narrative Reviews in Palliative Medicine Education. Palliative Medicine & Care: Open Access. 2020;7(1):1-22.

9. Mah ZH, Wong RSM, Seow REW, Loh EKY, Kamal NHA, Ong RRS, et al. A Systematic Scoping Review of Systematic Reviews in Palliative Medicine Education. Palliative Medicine & Care: Open Access. 2020;7(1):1-12.

10. France EF, Wells M, Lang H, Williams B. Why, when and how to update a meta-ethnography qualitative synthesis. Syst Rev. 2016;5:44.

11. Noblit GW HR. Meta-ethnography : synthesizing qualitative studies. Newbury Park: Sage Publications; 1988.

12. France EF, Uny I, Ring N, Turley RL, Maxwell M, Duncan EAS, et al. A methodological systematic review of meta-ethnography conduct to articulate the complex analytical phases. BMC Med Res Methodol. 2019;19(1):35.

13. Nasrallah S, Maytal G. Patient-Clinician Boundaries in Palliative Care Training: Identifying and Managing Boundary Crossings (517). Journal of Pain and Symptom Management. 2011;41(1):263-4.

14. Docherty SL, Miles MS, Brandon D. Searching for" the dying point:" providers' experiences with palliative care in pediatric acute care. Pediatric nursing. 2007;33(4).

15. Vig EK, Foglia MB. The steak dinner--a professional boundary crossing. J Pain Symptom Manage. 2014;48(3):483-7.

16. Schenell R, Ozanne A, Strang S, Henoch I. Balancing between maintaining and overriding the self: Staff experiences of residents' self‐determination in the palliative phases. International journal of older people nursing. 2019;14(4):e12255.

17. Sarraf-Yazdi S, Teo YN, How AEH, Teo YH, Goh S, Kow CS, et al. A Scoping Review of Professional Identity Formation in Undergraduate Medical Education. J Gen Intern Med. 2021;36(11):3511-21.
